# Commentary: A Neural Mechanism of Social Categorization

**DOI:** 10.3389/fnins.2019.00368

**Published:** 2019-04-17

**Authors:** Xiaoming Jiang, Ryan Sanford

**Affiliations:** ^1^Department of Psychology, School of Humanities, Tongji University, Shanghai, China; ^2^Department of Biomedical Engineering, McGill University, Montréal, QC, Canada

**Keywords:** social categorization, hand tracking, multivoxel pattern analysis, neural representational similarity analysis, right fusiform gyrus, dorsal anterior cingulate cortex

In social interactions we are constantly categorizing objects and people. While social categories (e.g., race) are sometimes ambiguous, the categorization process seems to be immediate and effective. Our brains are organized hierarchically to achieve efficient categorization. A dynamic interactive model considers social categorization as an outcome of the dynamic interplay between bottom-up perceptual representation and top-down impact of prior social knowledge paired with a category (Freeman and Johnson, 2016). This model specifies that the role of the fusiform gyrus (FG) is to represent visual features. The predictions based on the categorical knowledge from visual characteristics (i.e., stereotypical associations) are implemented in the orbital frontal cortex (OFC), which modulates the representations formed in the FG (Stolier and Freeman, 2016).

Stolier and Freeman investigated coactivation of opponent categories in the right FG (rFG) by calculating the neural pattern similarity of each atypical trial with the average neural pattern of its opponent category (e.g., similarity between an atypical male and average typical females). Stolier and Freeman (2017) increasing similarity was associated with larger hand trajectory deviation en route to the final category, suggesting coactivation of opponent categories in the rFG. Comparing BOLD responses between atypical and typical categories revealed activations in the presupplementary motor area and dorsal anterior cingulate cortex (pre-SMA/dACC). Stronger responses in the pre-SMA/dACC were associated with larger trajectory deviation. The authors concluded that processing opponent categories coactivate and compete in the rFG, while the competition is resolved into a stable categorization through the pre-SMA/dACC. Although this work provided compelling evidence that social categorization accomplished through dynamic competition between coactivated categories, this model may not be complete as additional factors were not considered.

Hand tracking paradigm reflects dynamic updates of a decision process as the hand's attraction toward each category provides an index regarding the extent of its activation. However, hand attraction may also be spontaneously guided through perceiver's bias toward “in-group” faces (Lazerus et al., 2016), or by attentionally-significant or motivationally-relevant faces (Ito and Urland, 2003; Kawakami et al., 2014). In addition, while the preferential bias enhances activity in the fusiform area (Van Bavel et al., 2011), it remains unclear whether the bias influences the neural representation in the rFG. Previous work has demonstrated that an individuals' hand trajectory was initially drawn toward more intuitive (i.e., familiar) categories, even when these categories were wrong (Traver et al., 2016). This means that the activation in the pre-SMA/dACC may reflect, at least in part, inhibition of strong tendencies toward a familiar category (e.g., a White perceiver judging an atypical Black face mixed with White facial cues). The response cost is not expected to occur when the final response is a familiar category but should arise for unfamiliar categories, affecting the involvement of pre-SMA/dACC to release the load of inhibition. The association between the neural pattern in the rFG and pre-SMA/dACC may occur when the inhibitory process is demanded. A trial-by-trial analysis that couples the pre-SMA/dACC activity and neural pattern in the rFG should be performed separately for atypical faces with different final responses and the neural coupling should be examined to assess the impact from perceivers' preferential biases. As compared with trials when the final category is familiar, the neural coupling is likely to be reduced for trials when the perceiver has to overcome spontaneous biases. As the unfolding of the trials, the spontaneous tendency to respond to the familiar category may weaken and the neural coupling may strengthen as the arising cost may be increasingly contributed by the co-activation of the opponent category. Other approaches to track the perceiver's anticipatory looks during the categorization process may be useful to monitor when a response suffers from spontaneous attractions.

Perceivers activate relevant stereotypes associated with a category in a quick-and-automatic fashion. Multiple social categories are processed interdependently if stereotypes related to categories overlap. For instance, the Asian race is commonly linked with femininity, meaning that categorizing a typical Asian-male face as male may be hindered. Stolier and Freeman minimized such stereotype-driven biases by collapsing gender when the neural pattern of race categorization was classified and vice versa. However, the relation between the trajectory deviation and neural pattern in the OFC (Shkurko, 2013) should be further examined to verify if the coactivation of the opposite category depends on top-down modulation of the representation (e.g., attentional coordination of multiple sources; Kveraga et al., 2007). Another important expansion of this model relates to how the neural pattern in the rFG is biased by contextual cues from vocal modalities, which can be sex/race-typical or -atypical (Freeman and Ambady, 2011). Social categories of faces paired with different voices can be highlighted by the neural pattern in the rFG and its connectivity with regions representing perceptual variances in human voice or cross-modal integration (Latinus and Belin, 2011).

The function of the pre-SMA/dACC in social categorization remains a large topic of debate. For instance, the dACC was reported to be activated when participants passively viewed stereotypically incongruent faces (e.g., happy Black male; Hehman et al., 2014). While the pre-SMA/dACC can be generally involved in conflict-monitoring, such responses can be triggered by enhanced cognitive control to monitor a state of uncertainty or co-existed competing categories. To resolve the possibilities, additional work should monitor the hand trajectory and analyze the BOLD response in the pre-SMA/dACC when participants categorize an atypical face that is mixed with irrelevant facial cues (e.g., a mixed White-Asian face in the White-Black categorization task). The strong association between pre-SMA/dACC and the cue relevance could be predicted by co-existed categories but not by the state of uncertainty. Further investigations are warranted to disentangle the role of the pre-SMA/dACC in social situations where categorization is done implicitly or explicitly for social inference.

This commentary points out some novel avenues toward a true characterization of neural mechanism of social categorization process with combined hand tracking and neuroimaging techniques. Additional work investigating perceiver's individual differences in their preferential bias and experiences toward certain categories are warranted. In addition, the neural coupling between rFG and pre-SMA/dACC and the association between neural responses and hand trajectory deviation in the presence of biases should be further investigated. Such investigations will provide a clearer understanding into the role of rFG and pre-SMA/dACC in social categorization. Moreover, little is known about the causal influence of top-down mechanism on the social categorization process. Further investigations are required to improve the understanding of the role of the OFC in social categorization. In conclusion, while this work provided compelling evidence that social categorization is accomplished through dynamic competition in the rFG with assistance from the pre-SMA/dACC, additional work is required to assess the model generalizability and provide a clearer understanding into neural mechanisms that underlie social categorizations, especially in the presence of biases and perceiver stereotypes.

## Author Contributions

XJ and RS both made substantial intellectual contributions to the manuscript and approved the final version for publication.

### Conflict of Interest Statement

The authors declare that the research was conducted in the absence of any commercial or financial relationships that could be construed as a potential conflict of interest.
